# Ocean acidification changes the vertical movement of stone crab larvae

**DOI:** 10.1098/rsbl.2019.0414

**Published:** 2019-12-11

**Authors:** Philip M. Gravinese, Ian C. Enochs, Derek P. Manzello, Robert van Woesik

**Affiliations:** 1 Mote Marine Laboratory, Fisheries Ecology and Enhancement, 1600 Ken Thompson Way, Sarasota, FL 34236, USA; 2 Florida Institute of Technology, Institute for Global Ecology, 150 W. Univ. Blvd., Melbourne, FL 32901, USA; 3 Atlantic Oceanographic and Meteorological Laboratories, National Oceanic and Atmospheric Administration, 4301 Rickenbacker Causeway, Miami, FL 33149, USA

**Keywords:** pH, climate change, crustacean, larval transport, elevated temperature

## Abstract

Anthropogenic activities are increasing ocean temperature and decreasing ocean pH. Some coastal habitats are experiencing increases in organic runoff, which when coupled with a loss of vegetated coastline can accelerate reductions in seawater pH. Marine larvae that hatch in coastal habitats may not have the ability to respond to elevated temperature and changes in seawater pH. This study examined the response of Florida stone crab (*Menippe mercenaria*) larvae to elevated temperature (30°C control and 32°C treatment) and CO_2_-induced reductions in pH (8.05 pH control and 7.80 pH treatment). We determined whether those singular and simultaneous stressors affect larval vertical movement at two developmental stages. Geotactic responses varied between larval stages. The direction and rate of the vertical displacement of larvae were dependent on pH rather than temperature. Stage III larvae swam upwards under ambient pH conditions, but swam downwards at a faster rate under reduced pH. There was no observable change in the directional movement of Stage V larvae. The reversal in orientation by Stage III larvae may limit larval transport in habitats that experience reduced pH and could pose challenges for the northward dispersal of stone crabs as coastal temperatures warm.

## 1. Introduction

Increasing atmospheric CO_2_ concentrations are warming the atmosphere and the ocean, and are causing a decline in ocean pH. Ocean temperatures are expected to increase by 2–4°C, and ocean pH is expected to decrease by 0.10–0.41 units by the end of the century [1,2]. Additionally, some coastal habitats are experiencing increased runoff and eutrophication, which amplifies pH variability [3–9]. In combination, these stressors can affect the development, behaviour, growth and survival of marine species, particularly during sensitive larval stages [10–12].

The distribution of most benthic marine populations is dependent on dispersal by planktonic larvae. Surface currents transport larvae away from hatching sites, although some larvae (e.g. brachyuran crustaceans) are capable of vertical migrations, which can adjust their horizontal transport when currents are depth stratified [13–19]. These vertical migrations are triggered by responses to biotic cues, but also abiotic stimuli, such as gravity [14–16], light, pressure [14,17,18] and pH [11]. Some marine species experience an impaired ability to orient to specific stimuli during exposure to reduced pH. For example, under reduced pH, hermit crabs struggle to locate prey [20] and shrimp display decreases in swimming ability [21]. Brachyuran crustacean larvae, which often rely on exogenous stimuli to direct vertical swimming, may be impacted by stressors like ocean acidification and elevated temperature.

Here, we examine the impact of these two stressors on the larval behaviour of the Florida stone crab, *Menippe mercenaria*. The stone crab fishery occurs throughout the southeastern United States and has an annual value of ∼$25–30 million in Florida [22]. Since 2000, the annual stone crab harvest has declined from 3.5 to 2.7 million pounds of claws per year [23]. Stone crab larvae take 20–30 days to complete development within coastal habitats [22–26]. Early-stage stone crab larvae exhibit vertical swimming behaviours in response to gravity, hydrostatic pressure and light that promote a relatively shallower depth distribution, whereas late-stage larvae reverse their vertical swimming response to the aforementioned cues resulting in a deeper distribution [26].

Land-use changes in Florida are increasing runoff and accelerating acidification in some coastal habitats (Tampa Bay ranges from 7.90 to 8.40; [9]). Some stone crab habitats (e.g. the Florida Keys) have also experienced an increase in temperature over the past century, which is problematic for a species already living close to its upper thermal limit [27]. Reductions in pH could disrupt enzymes and hormones necessary for moulting, whereas temperature increases can accelerate metabolism and growth, and destabilize proteins and enzymes [28–32]. Although stone crabs live in environments that experience variable temperature and carbonate chemistry (28.2–31.8°C; *p*CO_2_: 320–596 µatm during this study), their larvae are sensitive to pH, as a single stressor, which reduced hatching and survivorship by 28% and 37%, respectively [12,25]. Simultaneous exposure to reduced pH and elevated temperature was even more drastic, with 80% larval mortality [12].

Our study is the first to test the hypothesis that elevated temperature and reduced pH conditions will alter the vertical swimming behaviour of larval crustaceans using the Florida stone crab as a model example.

## 2. Material and methods

### (a) Experimental design

Ovigerous stone crabs (*n* = 25) were collected by the Florida Fish and Wildlife Conservation Commission using commercial traps near Pavilion Key (25° 69.79 N, 85° 35.51 W) during the summer of 2015. Crabs were maintained in control conditions until hatching. The experiment measured changes in larval vertical swimming behaviour after rearing larvae in the treatment conditions (table 1). Temperature was set at 30°C (control) and 32°C (elevated). The control was based on the Long Key C-MAN station in Florida Bay and corresponded to the mean summer temperature at the collection site [34]. The elevated temperature was based on the lower end of the sea-surface projections for 2100 (RCP-8.5) and corresponded to the upper historical mean summer sea-surface temperature (National Buoy Center: LONF1) within the Florida Keys [1,2]. The control pH (8.05) was based on pH at the collection site (table 1). The reduced pH treatment targeted conditions projected by the IPCC RCP-8.5 model for 2100 (pH 7.80; [1]).

**Table 1.** Mean daily (±s.d.) seawater carbonate chemistry, temperature and salinity. The treatment conditions were monitored for total alkalinity (A_T_) and pH_total_ during experimentation and *p*CO_2_ was derived from CO2SYS [33] (*n* = 52). Field samples (*n* = 10) were collected during the day between 08.00 and 12.00. Field samples were tested for A_T_ and dissolved inorganic carbon (DIC), while the pH_total_ and *p*CO_2_ were estimated using CO2SYS. The mean field DIC was 2104.0 µmol kg^−1^ ± 34.0. The change in the carbonate parameters between experimental treatment analyses and field sample analyses was the result of the DIC analyzer malfunctioning during experimentation.

treatments	temperature (°C)	A_T_ (µequiv kg^−1^)	pH_total_	*p*CO_2_ (µatm)	salinity
control	30.0 ± 0.2	2286.0 ± 36.7	8.05 ± 0.02	461.7 ± 35.5	37.7 ± 0.47
reduced pH	29.9 ± 0.3	2285.9 ± 34.7	7.78 ± 0.06	966.0 ± 157.9	37.7 ± 0.46
elevated temperature	31.9 ± 0.1	2282.6 ± 34.3	8.00 ± 0.02	571.7 ± 38.3	37.9 ± 0.50
reduced pH + elevated temperature	31.8 ± 0.2	2285.9 ± 35.1	7.74 ± 0.05	1137.3 ± 140.8	37.9 ± 0.51
field site	29.8 ± 0.4	2462.4 ± 28.8	8.04 ± 0.05	428.9 ± 72.6	34.9 ± 0.81

Seawater carbonate chemistry manipulations adhered to ocean acidification best practices [12,35]. All experimental chambers were monitored for total alkalinity (A_T_) and pH_total_. To avoid shocking the larvae, temperature and pH were gradually adjusted to the desired treatment over the first approximately 5 days of each experiment, which represents approximately 20% of the larval duration [12]. Stage I and II larvae never experienced the full experimental treatment conditions and were not used in the experiments. Experiments were performed on Stage III and V larvae to make comparisons to previously published work [26,36]. Stage IV larvae were not used in the experiments because of logistical challenges associated with performing multiple behavioural experiments on subsequent larval stages throughout their development. Larvae were reared *en masse* following procedures described in [12]. Experiments used larvae from independent broods (i.e. replicates). Each larval rearing chamber was independently controlled for temperature in a digitally controlled water bath that was independent from the other treatment combinations. Details of the experimental system, seawater manipulation and behavioural experiments are provided in the electronic supplementary material.

### (b) Geotaxis and larval swimming

The larval (*n* = 10 per treatment per brood) geotactic responses from replicate broods (Stage III = 7 broods; Stage V = 5 broods) were monitored for directional swimming among the treatments, according to established methods [26]. Larvae were randomly selected and checked for developmental stage prior to experimentation. Larval vertical movements were determined using a closed-circuit video system (Panasonic BP334 camera, Model-AG 1980 recorder) illuminated with far-red light (775 nm) [26,36,37]. An individual larva was pipetted into the centre of a clear acrylic tube (16 cm × 3 cm diameter), which was oriented horizontally in darkness. The tube was gently rotated 90° vertically to minimize fluid movement. Directional movements of individual larva were recorded throughout the middle 10 cm of the chamber until each larva moved out of the field of view, which on average ranged from 6 to 10 s. Larvae were only used once in any one of the treatments and discarded after experimentation. Larvae displaying a net upward response were recorded as ‘negatively geotactic’. A downward response was defined as a net displacement toward the chamber bottom. Larvae with no upward or downward displacement (after 10 s) were recorded as neutral. A permutation test was performed to test for differences in the geotaxic movement, with treatment as the main effect and brood as the blocking factor [38]. A mixed-effects model was used to test for differences among treatments using the individual larval swimming speeds. The individual larval swimming speed data were analysed in the model using positive and negative values, which represented upward or downward movements, respectively. The mixed-effects model was performed with treatment as a fixed factor and brood as a random effect nested within the treatment. All statistical analyses were performed using R v.3.6 [39].

## 3. Results

### (a) Geotaxis

**Figure 1.**
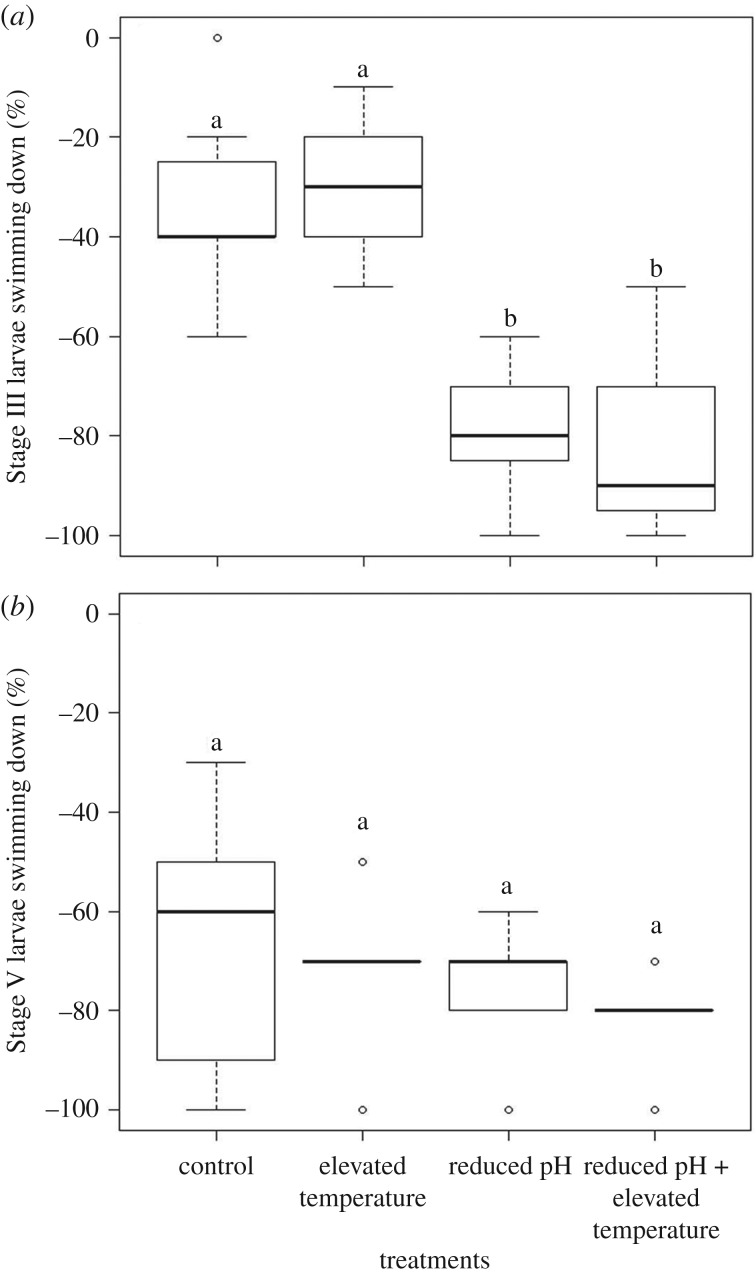
Box plot of the per cent of (*a*) Stage III and (*b*) Stage V larvae that swam down (%) among treatments. Different letters above the boxes indicate significant differences. Larvae that maintained the position upon stimulation (i.e. neutral swimming) were included in the upward responses. All trials were conducted in darkness. These data represent the distribution of averages within broods.

Stage III larvae raised in reduced pH showed a significant change in average swimming direction (temperature: *F*_1,6_ = 0.0, *p* = 1.0; pH: *F*_1,6_ = 85.5, *p* < 0.001). There was no statistically detectable interaction among the treatments (*F*_1,6_ = 0.29, *p* = 0.59); however, there was a marginally significant brood effect (*F*_6,18_ = 2.9, *p* = 0.03). The brood effect was driven by one brood's response in the reduced pH and elevated temperature treatment, and our low sample size (electronic supplementary material, figure S1). A larger proportion of Stage III larvae maintained their position or swam upward in the control pH treatments compared with individuals raised in low pH, which mostly moved downward (figure 1*a*). The experimental treatments did not have a significant effect on Stage V geotaxis (figure 1*b*; *p* > 0.05). Neutral responses in Stage III larvae ranged from 2 to 4% across treatments, whereas no Stage V larvae exhibited a neutral response (electronic supplementary material, table S1).

### (b) Larval swimming

**Figure 2.**
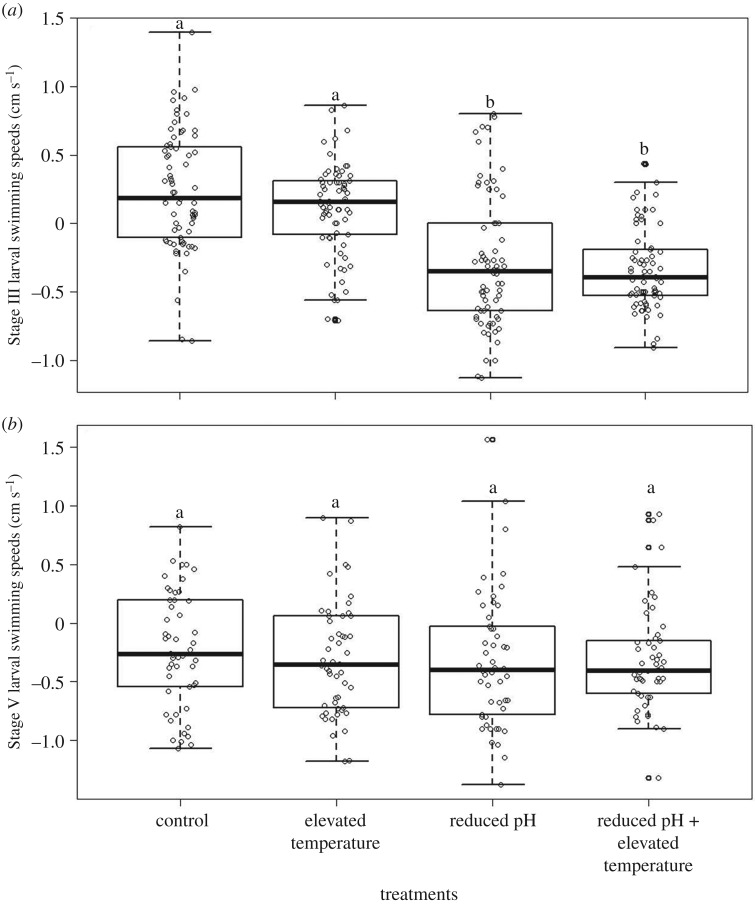
Box plot of the larval swimming speeds (cm s^−1^) for (*a*) Stage III and (*b*) Stage V larvae among treatments. Different letters above the bars indicate significant differences. Positive values represent upward movement and negative values represent downward movement. All trials were conducted in darkness. The open dots represent the distribution of individual larval swimming speeds within each treatment.

Stage III larvae exposed to reduced pH treatments moved downward significantly faster than larvae in the other treatments (*p* < 0.001; table 2 and figure 2; electronic supplementary material, figures S2 and S3). There was no statistically detectable effect of treatment in the Stage V swimming speeds (*p* > 0.05; table 2).

**Table 2.** Results of the linear mixed-effect analysis for the Stage III and V swimming speeds with brood as a random factor, which was nested within the treatment. Neutral responses were included in the analyses but only represented 2–4% of the total response in Stage III larvae and 0% of the response in Stage V larvae. Larvae that moved up were scored as positive swimming speeds, and larvae that moved down were scored as negative swimming speeds in the analysis.

source of variation	d.f.	*t*	*p*
*Stage III: swimming speed*			
temperature	18	−1.39	0.17
reduced pH	18	−5.71	<0.001
temperature + reduced pH	18	−6.07	<0.001
*Stage V: swimming speed*			
temperature	15	−0.58	0.56
reduced pH	15	−0.68	0.50
temperature + reduced pH	15	−0.65	0.52

## 4. Discussion

Stone crab larvae are known to exhibit vertical swimming behaviours to stimuli like gravity, pressure and light that promote depth regulation and facilitate dispersal [26]. Here, we show that stone crab larval swimming behaviours are affected by reduced pH. Our results suggest that low pH may change the vertical movement direction in some larval stages, which could alter dispersal in highly stratified waters.

Within our controls, a negative geotaxis response (i.e. upward swimming) in stone crab larvae is consistent with other crab species including *Callinectes sapidus* [40], *Rhithropanopeus harrisii* [37,41] and *Hemigrapsus sanguineus* [36] and positions newly hatched larvae in relatively shallow depths [26]. Some crustacean species (e.g. *H. sanguineus* and *M. mercenaria*) exhibit a reversal in their geotactic response during later larval stages, which has implications for larval transport in estuarine and coastal environments [13,14,26,36]. Stage III larvae exposed to only reduced pH showed the opposite directional response, indicating that pH but not temperature was interfering with vertical movement. The Stage III downward swimming speeds in reduced pH were slower than passively sinking individuals but faster than active larvae in ambient pH, indicating that larvae were controlling their descent or eliciting avoidance behaviour by attempting to move away from the reduced pH conditions. Reduced pH did not result in morphological abnormalities, changes in calcification or weight in larval stone crabs, suggesting that the descent was not related to factors impacting drag or buoyancy [12]. We acknowledge that our design could not account for fluid movement within the experimental chamber; therefore, some larvae may have been ‘stuck’ in boundary layers after rotation. However, given that most larvae remained close to the chamber's central axis, it is unlikely that chamber effects impacted larval movements.

The physiological mechanisms contributing to the change in the swimming direction were beyond the scope of this study; however, reduced pH acidifies crustacean haemolymph, which may have contributed to the change in swimming behaviour by altering enzymes and metabolism [42]. The inability to regulate acid–base balance under low pH has been shown to change swimming orientation in some coral-reef fish larvae [43–45]. The change in the directional response among Stage III larvae could also be the result of low pH impairing an alternative physiological mechanism that controls orientation. Orientation in many invertebrates is controlled by a calcareous statocyst, which is a sensory organ that forms during ontogeny [16,46]. The movement of the statolith triggers hairs that line the statocyst chamber, which provide sensory feedback to help the animal maintain equilibrium [47]. The observed change in swimming direction in Stage III larvae could be the result of statocyst degradation under reduced pH. Larval squid raised in reduced pH were reported to have abnormally shaped statoliths with the reduced surface area [48].

The change in stone crab larval swimming represents a short-term behavioural response to environmental stressors and may have implications for larval dispersal. The directional change in swimming could result in less precise depth regulation among Stage III larvae, resulting in a relatively deeper distribution, especially since downward vertical movements were faster in reduced pH. A deeper distribution in low pH could position individuals in slower currents restricting horizontal movement [13]. Consequently, these changes in vertical movement could keep larvae close to coastal habitats where predation is high and environmental conditions are not favourable for completing larval development. We caution that stone crab larval depth distributions should be confirmed via field sampling in habitats with different pH conditions.

Our study showed an ontogenetic shift in geotaxis, with Stage V larvae exhibiting behaviours promoting a deeper distribution, regardless of treatment. One possibility for observing no effect on Stage V larvae could be that this stage prefers to swim downward in ambient conditions [26]. Stone crab larvae can regulate their depth by kinetic responses to pressure changes [26]. By controlling their descent, the magnitude of pressure change experienced by the individual is less abrupt, allowing larvae to adjust their locomotor activity accordingly. The observed Stage V response could be the result of a controlled descent coupled with a positive barokinetic response, which may not be as sensitive to changes in pH as Stage III larvae.

Previous laboratory experiments demonstrated that elevated seawater temperature can affect brachyuran crustacean larval swimming by stimulating behaviours to avoid warmer surface waters [49]. Temperatures above an individual's upper limit can evoke a negative phototaxis, a positive geotaxis or inactivity, which all result in sinking [50]. Our study did not show any significant effects of temperature on larval swimming, suggesting some level of tolerance in larval stone crabs. The elevated temperature used in our study was conservative, based on the lower end of the sea-surface projections for 2100, and corresponded to the upper historical mean summer temperature for the study site. The temperature used may not have been elevated enough to elicit a measurable effect on larval movement.

Our results suggest that reduced pH may result in a relatively deeper distribution of Stage III stone crab larvae, which could reduce their short-term (days) transport capabilities by preventing their exposure to rapidly dispersing surface currents. Larval swimming responses to exogenous stimuli (e.g. gravity or light) mediate transport in coastal habitats and form the basis for conceptual models that describe how negatively buoyant crustacean larvae regulate their depth [14,16,50,51]. A change in the depth distribution of stone crab larvae may reduce dispersal and limit transport when exposed to reduced pH. These results have implications for the capacity of the species to adjust its northward geographical range as the oceans warm. If less precise depth regulation results in larvae being retained closer to near shore habitats, then individuals may be subject to greater predation pressure [52] and thermal extremes in shallower environments [12], all of which could further reduce larval supply [12]. To consider other aspects influencing dispersal, future studies should test larval responses to changes in light and pressure stimuli when exposed to reduced pH and elevated temperature conditions.

## Supplementary Material

Supplementary materials

## Supplementary Material

Water chemistry data

## Supplementary Material

Stage-III geotaxis data

## Supplementary Material

Stage-III swimming data all

## Supplementary Material

Stage-V geotaxis data

## Supplementary Material

Stage-V swimming data all

## Data accessibility

The datasets supporting this article have been uploaded as part of the electronic supplementary material.
