# Supplementary material for: Ocean acidification changes the vertical movement of stone crab larvae
Source: Biol Lett. 2019 Dec 11;15(12):20190414. doi: 10.1098/rsbl.2019.0414 (PMC6936016; doi:10.1098/rsbl.2019.0414)
Supplement: Supplementary materials [file rsbl20190414supp1.docx]

**Supplementary Material**

**Ocean acidification changes the vertical movement of stone crab larvae**

Philip M. Gravinese^a,b*^, Ian C. Enochs^c^, Derek P. Manzello^c^, and Robert van Woesik^a^

^a^Florida Institute of Technology, Institute for Global Ecology, 150 W. Univ. Blvd. Melbourne, FL 32901, United States of America

^b^Mote Marine Laboratory, Fisheries Ecology and Enhancement, 1600 Ken Thompson Way, Sarasota, FL 34236, United States of America

^c^Atlantic Oceanographic and Meteorological Laboratories, National Oceanic and Atmospheric Administration, 4301 Rickenbacker Causeway, Miami, Florida, 33149, United States of America

⃰**Corresponding author:** [**pgravinese@mote.org**](mailto:pgravinese@mote.org)

**Methodology**

***Study site, collection, and maintenance of experimental animals***

Ovigerous female crabs were collected by Florida Fish and Wildlife using commercial stone crab traps, 17 km from the coast in Florida Bay, Florida. All crabs were immediately transported back to NOAA’s Ocean Acidification laboratory and were maintained in our control seawater conditions until larval release. Upon larval release, newly hatched larvae were randomly assigned to each of the experimental treatments described below. Larvae from the same brood were divided among the treatments levels throughout all experiments and brood served as the unit of replication throughout these experiments.

***Experimental design and seawater chemistry manipulation***

Geotactic swimming experiments consisted of two treatments: 1) temperature, and 2) pH, each with two levels, resulting in a total of four different treatment combinations. The two temperature levels were set at 30°C and 32°C. The ambient conditions corresponded to levels at the site of ovigerous female collection (Table 1 in manuscript).

The targeted seawater control pH levels were achieved by passing seawater through a sand filter and a 100 mm mesh filter prior to being pumped into a holding reservoir, where seawater was subsequently aerated to ~450 µatm. The CO_2_ enrichment of treatment seawater was achieved by pumping seawater into a downstream treatment reservoir (1800 L), where pure CO_2_ gas and air was added using venturi injectors and mass flow controllers (MFC; SmartTrak 100, Sierra). Control and reduced pH seawater was then pumped into each of the separate experimental aquaria (7.5 L). The temperature within each experimental aquaria was regulated using heaters and temperature probes that were constantly monitored and maintained by a digital feedback system of AquaControllers (Apex, Neptune Systems). To avoid shocking the larvae, the use of MFCs and the digitally controlled temperature system allowed for a gradual adjustment of temperature and pH to the desired treatment set points over the first 5 days (“ramp-up period”) of each experiment. The experimental conditions were ramped up at ~200 µatm and ~0.4℃ per day to the desired treatment levels similar to (Gravinese et al. 2018). Stage-I and stage-II larvae never experienced the full experimental treatment conditions due to the gradual ramping up to experimental set points, and therefore were not used in the experimental analyses. We also did not use stage-IV larvae due to the logistical challenges associated with conducting multiple behavioral experiments on subsequent larval stages on multiple treatments. The target larval stages used in these experiments also allowed for making comparisons to other brachyuran crustacean swimming studies using similar larval stages (see Park et al. 2004, Gravinese 2018).

***Ocean acidification seawater chemistry***

The carbonate chemistry conditions in each treatment were monitored by collecting seawater samples from both the holding reservoirs and from each experimental aquaria in 150 mL borosilicate bottles, and were immediately fixed with 100 mL of saturated mercuric chloride. Carbonate parameters (Total alkalinity and total pH) were monitored every other day during the first week of the experiment, and every ~3–5 days thereafter. All seawater samples were taken between 12:00–14:00 for carbonate chemistry analyses. Total alkalinity (A_T_) was measured at NOAA's Atlantic Oceanographic and Metrological Ocean Acidification Laboratory using Apollo SciTech instruments (AS-ALK2) as described by Enochs et al. (2015). Alkalinity was checked for accuracy with certified reference materials (batch range 138–144; Dickson et al., 2003, Scripps Institution of Oceanography, La Jolla, CA) according to ocean acidification best practices. The total pH within each experimental aquarium was measured daily using a handheld pH meter (Oakton) and Ross electrode (Orion 9102BWNP; Thermoscientific). The pH electrodes were calibrated daily using Tris buffer. Temperature and salinity of each experimental aquarium were also monitored twice daily throughout all experiments (Orion Ecostar). The carbonate chemistry of seawater samples collected at the site of ovigerous female collection were analyzed for dissolved inorganic carbon and A_T_. Dissolved inorganic carbon (DIC) and A_T_ of field samples was analyzed at NOAA’s Atlantic Oceanographic and Metrological Ocean Acidification Laboratory using Apollo SciTech instruments (AS-ALK2 and AS-C3, respectively). Preservation of the collected field samples was similar to laboratory samples. Collection of field samples allowed us to confirm the control conditions were within the range of the *p*CO_2_ at field collection sites. All field samples were collected between 8:00–12:00 (N = 10) during the experimental season and all control/ambient *p*CO_2_ levels were within ranges reported for other stone crab habitats within Florida (Millero et al., 2001; Dufroe, 2012).

***Larval Rearing***

After hatching, larvae from the same brood were transferred into each of the experimental treatment combinations (i.e., temperature and pH). Larvae were reared in 7.5 L containers (~250 individuals L^-1^), with 80% of the containers sides being composed of nylon mesh (24 cm diameter x 24 cm depth; 190 µm mesh) and a stocking density ~250 individuals L^-1^. Venturi pumps were positioned to facilitate water exchange across the mesh. Each larval rearing container was kept in its own digitally controlled water bath which was independent from the other treatment combinations and maintained at the set-points as previously described.

Prior to feeding larvae, *Artemia* were enriched with a lipid diet (Selco, Brine shrimp direct, UT) and fed enriched rotifers. Rotifers that were fed to *Artemia* were also enriched with a high protein and lipid diet (One Step, Rotigrow, CA). Prior to harvesting larvae for experiments, a subsample of 50 larvae was assessed to determine the developmental stage (Porter, 1960). We then randomly selected a new set of larvae for developmental stage and used the first ten larvae that were at the targeted developmental stage (i.e., stage-III or stage-V). Most larvae take about 10–12 days to reach stage-III and about 20–25 days to reach stage-V (Gravinese et al. 2018).
 Larvae were kept on a 14 h-light:10h-dark photoperiod that approximated conditions during the time of collection. Dead larvae and wastes were removed from each chamber twice daily to minimize the buildup of nutrients. Larvae selected for experiments were only used once and then discarded after experimentation.

***Sinking rates***

Stage-III and -V larvae (20 per treatment combination, n = 5 broods) were harvested and anesthetized using a 10% MgCl_2_ solution. After larvae were anesthetized, individual larvae were pipetted into a vertical acrylic chamber (21.6 x 8.3 x 8.3 cm) and allowed to sink for 10 cm. Sinking rates were calculated by determining the rate at which the anesthetized larvae traversed the 10 cm section of the chamber during each trial (Arana and Sulkin, 1993, Gravinese 2018). Passive sinking rates for both larval stages were analyzed for statistical differences among treatments using a Friedman test as the data did not meet the normality assumption for an analysis of variance with repeated measures. Pairwise comparisons among passive sinking rates and downward swimming responses within each treatment for stage-III larvae were compared using a Mann-Whitney test (Table S1).

***Geotaxis***

The geotactic responses of larvae from multiple broods (replicates; stage-III = 7 broods; stage-V = 5 broods) were monitored for upward or downward movement (%) among the experimental treatment combinations. The vertical movements of larvae were determined using a closed circuit video system (Panasonic BP334 B/W video camera attached to a Panasonic Model AG 1980 video recorder) illuminated with infrared light (775 nm). Individual larvae were placed in the center of a clear acrylic tube which was oriented horizontally and kept in dark conditions for a 2 minute acclimation period. The tube was gently and slowly rotated 90° vertically to minimize fluid movement, and the directional movements (up or down) of larvae were recorded (similar to Arana and Sulkin 1993 and Gravinese 2018). Larvae that did not move up or down (i.e., maintained position) were recorded as a neutral response after 10 seconds of observation. Downward and upward swimming speeds were determined by measuring the time that larvae took to move vertically within the chamber (either up or down). Geotaxis experiments were conducted haphazardly between 9:00–21:00 on the day of the experiment.

**Additional Results**

***(a) Geotaxis***

There was a marginally significant brood effect in the stage-III upward swimming response. The brood effect was likely driven by: 1) brood F’s response in the reduced pH and elevated temperature treatment and 2) our low sample size (Figure S1). The percentage of larvae moving up, down, or eliciting a neutral response is presented in Table S1.

**
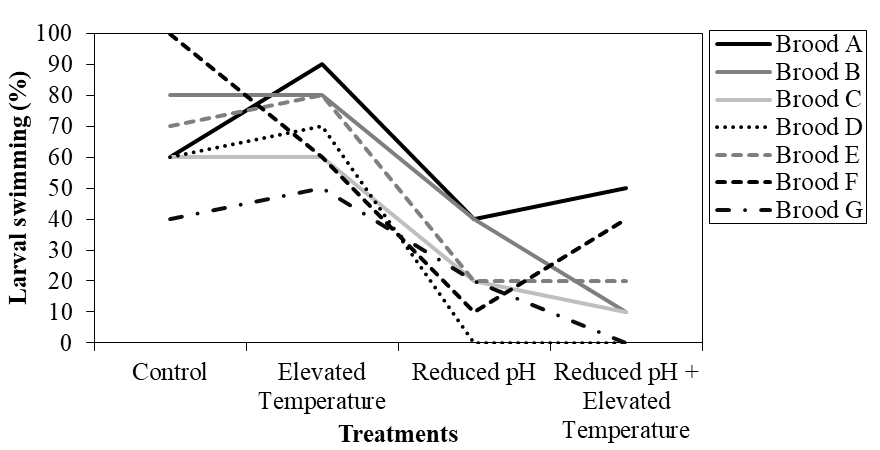
**

**Figure S1**. Interaction plot showing the brood specific responses for stage-3 larvae during the geotaxis experiment.

**Table S1**. Percentage of individual larvae moving up, down, or not moving in either direction (neutral) among the experimental treatments using larvae from 7 broods for stage-III and 5 broods for stage-V.

| Direction of Movement | Treatments | | | |
| --- | --- | --- | --- | --- |
| *Stage-III* | Control | Reduced pH | Elevated Temperature | Reduced pH + Elevated Temperature |
| Up | 65.7 | 21.4 | 70.0 | 18.6 |
| Neutral | 2.8 | 4.3 | 2.8 | 2.8 |
| Down | 31.4 | 74.3 | 27.1 | 78.6 |
| *Stage-V* |  |  |  |  |
| Up | 18.0 | 24.0 | 28.0 | 34.0 |
| Neutral | 0 | 0 | 0 | 0 |
| Down | 82.0 | 76.0 | 72.0 | 66.0 |

***(b) Passive sinking and swimming speeds***

The Friedman test showed no statistical differences in the stage-III passive sinking rates (Χ^2^ = 3.1, P = 0.38) or the stage-V passive sinking rates among treatments (Χ^2^ = 3.5, P = 0.32). Sinking rates were significantly faster than the downward swimming speeds during geotaxis trials (P< 0.001 for both stage-III and -V larvae Table S2). There were no significant differences among the stage-V downward swimming speeds (see main text for results). The individual larval swimming speeds for each brood across treatments are depicted in Figures S2 (stage-III larvae) and S3 (stage-V larvae). A mixed-effects model was used to test for differences among treatments using the individual larval swimming speeds. The mixed effects model was performed with individual swimming speed (positive for upward swimming or negative for downward swimming) as the dependent variable and treatment as the fixed factor. Brood was a random effect, which was nested within treatment. The model assumes the Kenward-Roger method for estimating degrees of freedom and was performed using the “lme4” and “afex” packages in R (Bates et al. 2015, Henrik et al. 2019). The Q-Q plots for the larval swimming speeds are also provided in Figures S4-S5. The lmer models are robust for the normality assumptions.

**Table S2:** Results of the pairwise comparisons among passive sinking rates and downward swimming responses within each treatment for stage-III larvae. A Mann-Whitney test was performed.

| **Treatment** | **Activity** | **N** | **Mean Rank** | **Test Statistic** | **P-value** |
| --- | --- | --- | --- | --- | --- |
| Control | Sinking  Swimming | 100  21 | 71.5 11.0 | U = 2100 | P < 0.001 |
| Reduced pH | Sinking  Swimming | 100  52 | 99.5 32.2 | U = 4903 | P < 0.001 |
| Elevated temperature | Sinking  Swimming | 100  19 | 69.5  10.0 | U = 1900 | P < 0.001 |
| Reduced pH + Elevated tempearature | Sinking  Swimming | 100  55 | 104.4  30.0 | U = 5390 | P < 0.001 |

**
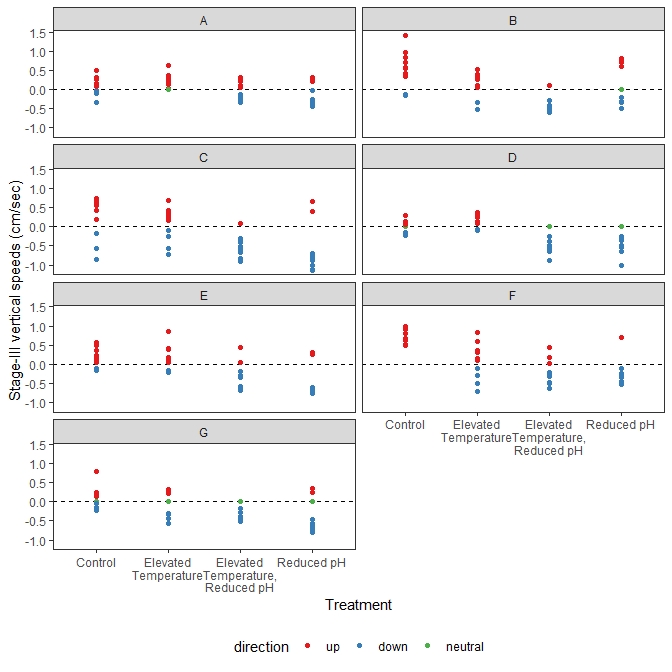
**

**Figure S2:** Stage-III individual larval vertical swimming speeds (cm sec^-1^) for each brood (letters A–G) across the experimental treatments. Positive values represent upward speeds and negative values represent downward speeds. Red dots represent upward moving larvae, blue dots represent downward moving larvae, and green dots represent larvae that elicited a neutral response.

**
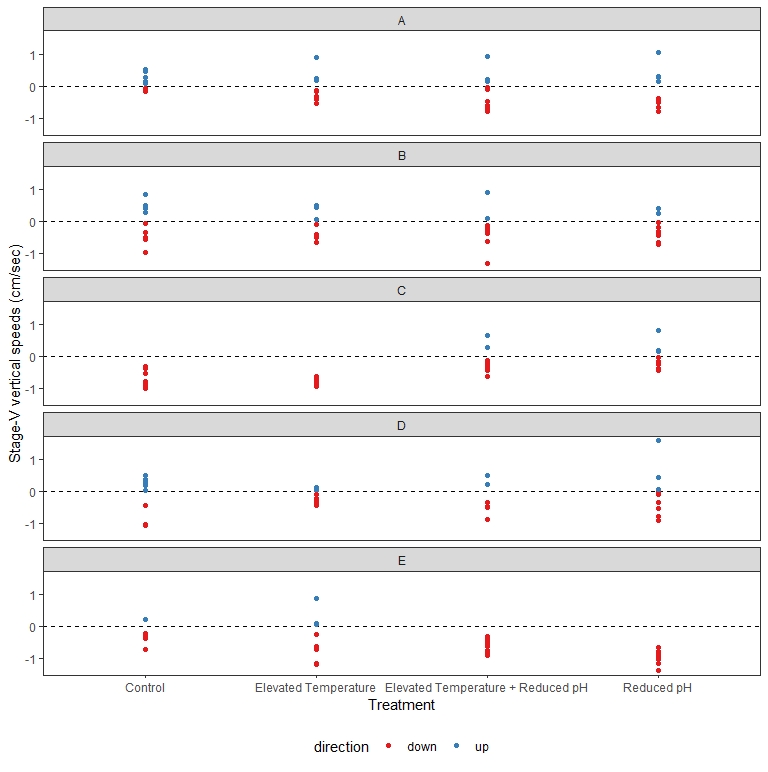
**

**Figure S3:** Stage-V individual larval vertical swimming speeds (cm sec^-1^) for each brood (letters A–E) across the experimental treatments. Positive values represent upward speeds and negative values represent downward speeds. Red dots represent upward moving larvae and blue dots represent downward moving larvae. There were no neutral responses observed in stage-V larvae.

**
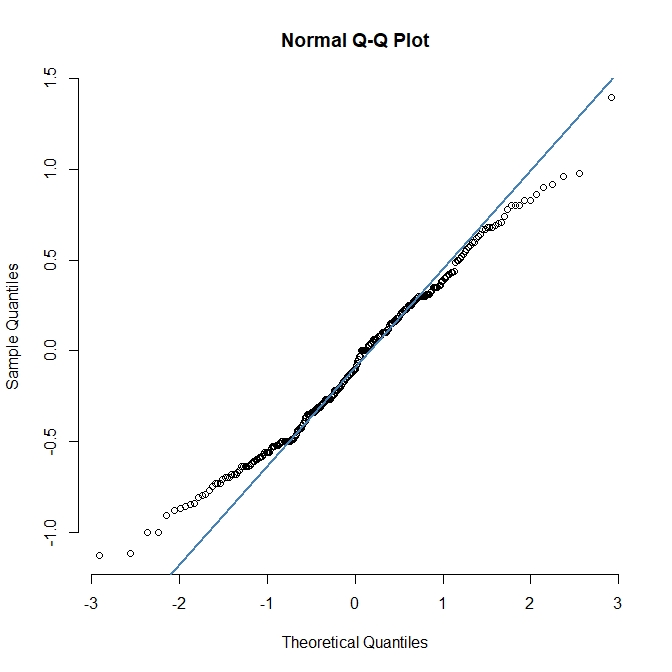
**

**Figure S4:** Q-Q plot for stage-III individual larval vertical swimming speeds (sample quantiles) vs the theoretical values. Positive values represent upward movement and negative values represent downward movement. The solid line passes through the first and third quantiles.

**
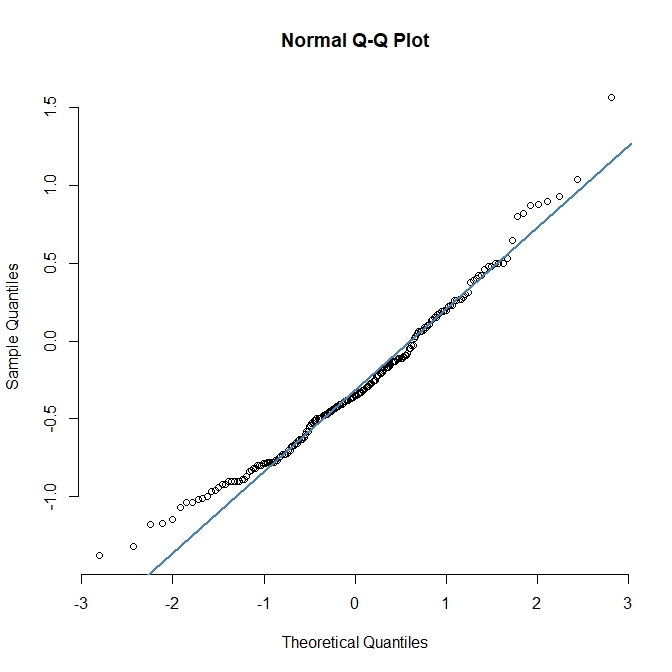
**

**Figure S5:** Q-Q plot for stage-V individual larval vertical swimming speeds (sample quantiles) vs the theoretical values. Positive values represent upward movement and negative values represent downward movement. The solid line passes through the first and third quantiles.

**Supplementary References**

Arana M, Sulkin SD. 1993. Behavioral basis of depth regulation in the first zoeal stage of the Pacific shore crab, *Hemigrapsus oregonensis* (Brachyura: Grapsidae). *Pac. Sci.* **47**, 256–262.

Bates D, Maechler M, Bolker B, Walker S. 2015. Fitting Linear Mixed-Effects Models Using lme4. *J. Stat. Softw*., 67(1), 1-48. doi:10.18637/jss.v067.i01.

Conover WJ, Iman RL. 1981. Rank transformations as a bridge between parametric and nonparametric statistics. *Am. Stat*. **35**, 124–129.

Dickson AG, Afghan JD, Anderson GC. 2003. Reference materials for oceanic CO_2_ analysis: a method for the certification of total alkalinity. *Mar. Chem*. **80**, 185–197.

Dufroe CM. 2012. Spatial and Temporal Variations in the Air-sea Carbon Dioxide Fluxes of Florida Bay. Ph.D. dissertation. University of South Florida, Tampa, FL,unpublished.

Enochs IC, Manzello DP, Donham EM, Kolodziej G, Okano R, Johnston L, Young C, Iguel J, Edwards CB, Fox MD, Valentino L. 2015. Shift from coral to macroalgae dominance on a volcanically acidified reef. *Nat. Clim. Change*. **5**(12):1083.

Felgenhauer BE, Abele LG. 1983. Ultrastructure and functional morphology of feeding and associated appendages in the tropical fresh-water shrimp *Atya innocuous* (Herbst) with notes on its ecology. *J. Crust. Biol*. **3**(3), 336–363.

Gravinese PM. 2018. Vertical swimming behavior in larvae of the Florida stone crab, *Menippe mercenaria. J. Plankton Res*. **40**(6), 627–642**.**

Gravinese PM, Enochs IC, Manzello DP, van Woesik R. 2018. Warming and *p*CO_2_ effects on Florida stone crab larvae. *Est. Coast. Shelf Sci.* **204**, 193–201.

Henrik S, Bolker B, Westfall J, Aust F, Ben-Shachar, MS. 2019. afex: Analysis of Factorial Experiments. R package version 0.25-1. <https://CRAN.R-project.org/package=afex>

Intergovernmental Panel on Climate Change (IPCC). 2013. The physical science basis. Working Group I contribution to the 5^th^ assessment report of the Intergovernmental Panel on Climate Change. Cambridge University Press.

Millero FJ, Hiscock WT, Huang F, Roche M, Zhang JZ. 2001. Seasonal variation of the carbonate system in. Florida Bay. *Bull. Mar. Sci*. **68**, 101–123.

NOAA National Data Buoy Center. <http://www.ndbc.noaa.gov/view_climplot.php?station1/4lonfl&meas1/4st>. (accessed 3, February 2016).

Porter HJ. 1960. Zoeal stages of the stone crab, *Menippe mercenaria* (Say). *Chesap. Sci.***1**, 168–177.

Park S, Epifanio CE, Grey EK. 2004 Behavior of larval *Hemigraspus sanguineus* (de Haan) in response to gravity and pressure. *J. Exp. Mar. Biol. Ecol*. **307**(2), 197–206.

R Development Core Team. 2016. R: A language and environment for statistical computing. R Foundation for Statistical Computing, Vienna, Austria, ISBN 3-900051-07-0. http://www.R-project.org.

**Statistical code**

**Geotaxis**

install.packages("lmPerm") *#install packages for permutation test*

library(lmPerm) *# allows for restricting the permutations within subjects*

***Stage-III and Stage-V Geotaxis***

z3geo<-read.csv("stage3_geotaxis.csv", header=T, na.strings=c(""))*# file name*

z5geo<-read.csv("stage5_geotaxis.csv", header=T, na.strings=c(""))*# file name*

summary(aovp(Up~Temp*CO2+Brood,data= z3geo,perm="Approximate", seqs =F,nCycle = 9999)) *# geotaxis analysis using the lmPerm package, allows for repeated #measures, brood is a blocking variable. This code if for stage-3 geotaxis*

summary(aovp(Up~Temp*CO2+Brood,data= z5geo,perm="Approximate", seqs =F,nCycle = 9999))*# geotaxis analysis using the lmPerm package, allows for repeated #measures, brood is a blocking variable. This code if for stage-5 geotaxis*

**Stage-III Swimming Speeds Code**

#*installing necessary packages*

install.packages("lme4")

install.packages("dplyr")

install.packages("languageR")

install.packages("afex")

library(lme4)

library(dplyr)

library(languageR)

library(afex)

*#File name*

stage3_indiv<-read.csv("Stage3_ALL.csv", header=T, na.strings=c(""))# file name

*#create a new variable for signed velocity*

*#with upward swimming coded as positive and downward as negative*

stage3_indiv = read.csv("Stage3_ALL.csv",stringsAsFactors = FALSE)%>%

mutate(

speed_full = speed*ifelse(direction=="up",1,-1),

direction = factor(direction,levels =c("up","down","neutral")),

Treatment = str_replace(Treatment,

pattern = "Elevated Temperature",

"Elevated\nTemperature"),

Treatment = str_replace(Treatment,pattern = ".\\+ ",

",\n"))

#*calculate a brood-level average vertical swimming rate for each treatment*

stage3_brood = stage3_indiv %>%

group_by(Brood, Treatment)%>%

summarize(speed_full = mean(speed_full),

speed_up = mean(speed_full[direction=="up"]),

speed_down = mean(speed_full[direction=="down"]))

#*individual-level model with two levels of random effects: a mean brood level, and a brood-within-treatment level*

stage3_indiv_speed <- lmer(speed_full ~ Treatment+(1|Brood/Treatment), REML = FALSE,

data = stage3_indiv)

summary(stage3_indiv_speed)

#*Q-Q plot for stage-III swimming speeds*

qqnorm(stage3_indiv$speed_full, pch = 1, frame = FALSE)

qqline(stage3_indiv$speed_full, col = "steelblue", lwd = 2)

**Stage-V Swimming Speeds Code**

*#File name*

stage5_indiv<-read.csv("Stage5_ALL.csv", header=T, na.strings=c(""))# file name

*#create a new variable for signed velocity,*

*#with upward swimming coded as positive and downward as negative*

Stage5_indiv = read.csv("Stage5_ALL.csv",stringsAsFactors = FALSE)%>%

mutate(

speed_full = speed_full*ifelse(direction=="up",1,-1),

direction = factor(direction,levels =c("up","down","neutral")),

Treatment = str_replace(Treatment,

pattern = "Elevated Temperature",

"Elevated\nTemperature"),

Treatment = str_replace(Treatment,pattern = ".\\+ ",

",\n"))

#*calculate a brood-level average vertical swimming rate for each treatment*

stage5_brood = stage5_indiv %>%

group_by(Brood, Treatment)%>%

summarize(speed_full = mean(speed_full),

speed_up = mean(speed_full[direction=="up"]),

speed_down = mean(speed_full[direction=="down"]))

#*individual-level model with two levels of random effects: a mean brood level, and a brood-within-treatment level*

stage5_indiv_speed <- lmer(speed_full ~ Treatment+(1|Brood/Treatment),REML = FALSE,data = stage5_indiv)
summary(stage5_indiv_speed)

#*Q-Q plot for stage-V swimming speeds*

qqnorm(stage5_indiv$speed_full, pch = 1, frame = FALSE)

qqline(stage5_indiv$speed_full, col = "steelblue", lwd = 2)

**Water Chemistry Data (post ramp-up conditions)**

| Treatment | Temperature (℃) | Salinity | pH_Total | Total alkalinity (µequiv kg^-1^) | *p*CO_2_ (µatm) | Date |
| --- | --- | --- | --- | --- | --- | --- |
| Control | 30.3 | 37.1 | 8.04 | 2269.0 | 483.70 | 5/26 |
| Control | 30 | 37.2 | 8.03 | 2263.3 | 490.59 | 5/26 |
| Control | 30.1 | 37.2 | 8.03 | 2290.2 | 491.50 | 5/26 |
| Control | 30.4 | 37 | 8.03 | 2254.3 | 496.22 | 5/26 |
| Control | 30 | 37 | 8.03 | 2316.9 | 495.67 | 5/31 |
| Control | 30.2 | 37.1 | 8.06 | 2314.3 | 459.87 | 5/31 |
| Control | 30 | 37.2 | 8.08 | 2316.5 | 438.02 | 5/31 |
| Control | 30.3 | 37 | 8.06 | 2317.7 | 458.27 | 5/31 |
| Control | 30.1 | 37.2 | 8.03 | 2301.8 | 498.82 | 6/5 |
| Control | 30.3 | 37.2 | 8.03 | 2332.3 | 504.59 | 6/5 |
| Control | 30.1 | 37.1 | 8.06 | 2332.6 | 466.65 | 6/5 |
| Control | 30.3 | 37 | 8.03 | 2334.1 | 502.66 | 6/5 |
| Control | 30.1 | 37.3 | 8.02 | 2338.4 | 516.16 | 6/13 |
| Control | 29.8 | 37.3 | 8.01 | 2334.3 | 522.69 | 6/13 |
| Control | 30 | 37.3 | 8.04 | 2330.4 | 483.73 | 6/13 |
| Control | 30 | 37.1 | 8.05 | 2319.0 | 472.31 | 6/13 |
| Control | 30.1 | 37.8 | 8.02 | 2209.5 | 492.47 | 6/16 |
| Control | 30.2 | 37.9 | 8.01 | 2213.1 | 505.57 | 6/16 |
| Control | 30 | 38 | 8.02 | 2209.7 | 487.02 | 6/16 |
| Control | 30 | 37.8 | 8.03 | 2206.5 | 466.15 | 6/16 |
| Control | 30.1 | 37.9 | 8.03 | 2261.7 | 485.97 | 6/21 |
| Control | 30.2 | 37.9 | 8.07 | 2246.5 | 429.53 | 6/21 |
| Control | 30.2 | 37.9 | 8.06 | 2249.8 | 443.37 | 6/21 |
| Control | 30 | 37.9 | 8.07 | 2252.5 | 433.16 | 6/21 |
| Control | 29.9 | 38.1 | 8.07 | 2272.0 | 426.77 | 6/26 |
| Control | 30 | 38.1 | 8.08 | 2275.9 | 422.28 | 6/26 |
| Control | 30 | 38.2 | 8.09 | 2269.3 | 411.33 | 6/26 |
| Control | 30.1 | 38.3 | 8.07 | 2268.6 | 427.76 | 6/26 |
| Control | 30.4 | 38.2 | 8.02 | 2305.9 | 508.19 | 7/8 |
| Control | 30 | 38.1 | 8.03 | 2304.2 | 489.49 | 7/8 |
| Control | 29.7 | 38.3 | 8.03 | 2316.6 | 492.51 | 7/8 |
| Control | 29.5 | 38.2 | 8.06 | 2309.6 | 443.79 | 7/8 |
| Control | 29.6 | 38.1 | 8.08 | 2331.7 | 429.23 | 7/11 |
| Control | 30 | 38.1 | 8.06 | 2330.5 | 454.24 | 7/11 |
| Control | 30.3 | 38.3 | 8.06 | 2334.1 | 458.38 | 7/11 |
| Control | 29.3 | 38.2 | 8.08 | 2328.4 | 416.27 | 7/11 |
| Control | 29.7 | 38.3 | 8.08 | 2259.5 | 416.76 | 7/16 |
| Control | 29.8 | 38.4 | 8.08 | 2258.9 | 419.82 | 7/16 |
| Control | 30.2 | 38.3 | 8.07 | 2263.1 | 433.17 | 7/16 |
| Control | 30 | 38.2 | 8.08 | 2260.6 | 413.14 | 7/16 |
| Control | 29.8 | 37.7 | 8.09 | 2262.9 | 410.57 | 7/27 |
| Control | 29.9 | 37.8 | 8.07 | 2281.2 | 438.30 | 7/27 |
| Control | 30.3 | 37.8 | 8.07 | 2272.6 | 437.24 | 7/27 |
| Control | 29.9 | 37.7 | 8.09 | 2269.2 | 402.80 | 7/27 |
| Control | 29.8 | 38.2 | 8.06 | 2265.2 | 442.95 | 7/30 |
| Control | 29.8 | 38.1 | 8.06 | 2272.4 | 436.41 | 7/30 |
| Control | 30.1 | 38.3 | 8.06 | 2274.2 | 449.20 | 7/30 |
| Control | 30 | 38.1 | 8.08 | 2269.4 | 425.85 | 7/30 |
| Control | 29.8 | 38.2 | 8.05 | 2275.5 | 455.29 | 8/4 |
| Control | 29.9 | 38.2 | 8.03 | 2350.4 | 501.06 | 8/4 |
| Control | 30.3 | 38.1 | 7.99 | 2297.1 | 555.16 | 8/4 |
| Control | 29.6 | 38.3 | 8.04 | 2279.3 | 469.26 | 8/4 |
| Elevated temperature | 31.9 | 37.7 | 8.03 | 2253.3 | 517.27 | 5/26 |
| Elevated temperature | 31.8 | 37 | 8.02 | 2263.0 | 539.56 | 5/26 |
| Elevated temperature | 31.7 | 37.2 | 8.00 | 2262.7 | 564.67 | 5/26 |
| Elevated temperature | 32 | 37 | 8.01 | 2264.3 | 557.15 | 5/26 |
| Elevated temperature | 31.8 | 37.1 | 8.03 | 2306.5 | 539.76 | 5/31 |
| Elevated temperature | 31.8 | 37.1 | 8.03 | 2315.3 | 539.42 | 5/31 |
| Elevated temperature | 31.8 | 37.1 | 8.03 | 2315.9 | 533.77 | 5/31 |
| Elevated temperature | 31.8 | 37.3 | 8.02 | 2317.0 | 544.83 | 5/31 |
| Elevated temperature | 31.8 | 37.1 | 7.99 | 2296.0 | 586.54 | 6/5 |
| Elevated temperature | 31.7 | 37.1 | 7.99 | 2294.2 | 592.15 | 6/5 |
| Elevated temperature | 31.8 | 37.3 | 7.99 | 2333.5 | 597.49 | 6/5 |
| Elevated temperature | 31.9 | 37.1 | 8.00 | 2331.6 | 588.15 | 6/5 |
| Elevated temperature | 31.8 | 37.4 | 7.99 | 2322.1 | 592.91 | 6/13 |
| Elevated temperature | 31.8 | 37.3 | 7.99 | 2335.5 | 599.69 | 6/13 |
| Elevated temperature | 31.7 | 37.2 | 7.99 | 2332.2 | 591.76 | 6/13 |
| Elevated temperature | 31.7 | 37.2 | 8.00 | 2322.3 | 574.88 | 6/13 |
| Elevated temperature | 31.9 | 37.9 | 7.98 | 2208.8 | 587.53 | 6/16 |
| Elevated temperature | 31.7 | 38.2 | 7.97 | 2211.4 | 589.28 | 6/16 |
| Elevated temperature | 31.7 | 38.1 | 7.99 | 2210.2 | 569.01 | 6/16 |
| Elevated temperature | 31.7 | 38.1 | 7.98 | 2207.4 | 573.49 | 6/16 |
| Elevated temperature | 31.8 | 38.1 | 7.98 | 2267.5 | 597.56 | 6/21 |
| Elevated temperature | 31.8 | 38.1 | 7.98 | 2261.6 | 599.59 | 6/21 |
| Elevated temperature | 31.9 | 38.1 | 8.01 | 2249.8 | 553.37 | 6/21 |
| Elevated temperature | 32 | 38 | 8.01 | 2270.3 | 552.44 | 6/21 |
| Elevated temperature | 32 | 38.3 | 8.01 | 2269.3 | 546.01 | 6/26 |
| Elevated temperature | 31.7 | 38.1 | 8.02 | 2270.1 | 537.23 | 6/26 |
| Elevated temperature | 31.8 | 38.3 | 8.03 | 2277.4 | 526.90 | 6/26 |
| Elevated temperature | 32 | 38.3 | 8.03 | 2261.2 | 523.34 | 6/26 |
| Elevated temperature | 32 | 38.3 | 7.96 | 2306.5 | 646.32 | 7/8 |
| Elevated temperature | 31.9 | 38.3 | 7.98 | 2309.5 | 611.62 | 7/8 |
| Elevated temperature | 31.8 | 38.2 | 7.97 | 2308.6 | 632.39 | 7/8 |
| Elevated temperature | 32.3 | 38.4 | 7.94 | 2310.3 | 681.75 | 7/8 |
| Elevated temperature | 32 | 38.2 | 8.00 | 2333.7 | 589.42 | 7/11 |
| Elevated temperature | 31.9 | 38.3 | 8.00 | 2332.4 | 582.16 | 7/11 |
| Elevated temperature | 32.1 | 38.4 | 7.99 | 2333.9 | 598.86 | 7/11 |
| Elevated temperature | 32.1 | 38.3 | 8.00 | 2329.2 | 588.92 | 7/11 |
| Elevated temperature | 31.8 | 38.4 | 7.99 | 2252.7 | 572.99 | 7/16 |
| Elevated temperature | 32 | 38.4 | 7.98 | 2259.2 | 594.82 | 7/16 |
| Elevated temperature | 32 | 38.4 | 8.01 | 2252.3 | 550.82 | 7/16 |
| Elevated temperature | 31.6 | 38.4 | 8.03 | 2265.7 | 517.04 | 7/16 |
| Elevated temperature | 31.8 | 37.8 | 8.03 | 2265.5 | 515.96 | 7/27 |
| Elevated temperature | 31.7 | 37.9 | 8.03 | 2275.0 | 523.31 | 7/27 |
| Elevated temperature | 31.9 | 37.9 | 8.01 | 2271.0 | 550.93 | 7/27 |
| Elevated temperature | 31.8 | 37.8 | 8.04 | 2272.3 | 506.61 | 7/27 |
| Elevated temperature | 31.6 | 38.3 | 8.00 | 2271.6 | 557.89 | 7/30 |
| Elevated temperature | 31.7 | 38.3 | 8.00 | 2268.5 | 565.91 | 7/30 |
| Elevated temperature | 31.9 | 38.2 | 8.01 | 2272.9 | 554.07 | 7/30 |
| Elevated temperature | 31.8 | 38.3 | 8.03 | 2275.6 | 513.21 | 7/30 |
| Elevated temperature | 31.8 | 38.2 | 7.94 | 2274.6 | 663.80 | 8/4 |
| Elevated temperature | 31.8 | 38.3 | 7.97 | 2276.7 | 612.11 | 8/4 |
| Elevated temperature | 32 | 38.4 | 7.97 | 2276.4 | 616.45 | 8/4 |
| Elevated temperature | 31.9 | 38.4 | 8.00 | 2272.0 | 569.04 | 8/4 |
| Reduced pH | 30 | 37.2 | 7.83 | 2265.3 | 833.02 | 5/26 |
| Reduced pH | 30.2 | 37.1 | 7.80 | 2269.8 | 921.25 | 5/26 |
| Reduced pH | 30.2 | 37.1 | 7.82 | 2262.9 | 870.42 | 5/26 |
| Reduced pH | 30.4 | 37.1 | 7.81 | 2254.9 | 897.20 | 5/26 |
| Reduced pH | 30 | 37.2 | 7.72 | 2306.2 | 1135.98 | 5/31 |
| Reduced pH | 30.1 | 37.1 | 7.72 | 2309.0 | 1133.45 | 5/31 |
| Reduced pH | 30 | 37.1 | 7.74 | 2305.5 | 1070.47 | 5/31 |
| Reduced pH | 29.8 | 37.2 | 7.72 | 2319.6 | 1142.20 | 5/31 |
| Reduced pH | 30.2 | 37.1 | 7.68 | 2296.5 | 1272.70 | 6/5 |
| Reduced pH | 30 | 37.2 | 7.71 | 2301.3 | 1171.40 | 6/5 |
| Reduced pH | 30 | 37.1 | 7.69 | 2328.7 | 1244.35 | 6/5 |
| Reduced pH | 30.2 | 37.2 | 7.66 | 2326.5 | 1344.05 | 6/5 |
| Reduced pH | 30.3 | 37.2 | 7.76 | 2342.0 | 1042.71 | 6/13 |
| Reduced pH | 29.8 | 37.2 | 7.75 | 2341.1 | 1055.91 | 6/13 |
| Reduced pH | 30 | 37.1 | 7.77 | 2343.7 | 1013.75 | 6/13 |
| Reduced pH | 30.1 | 37.2 | 7.79 | 2329.8 | 973.14 | 6/13 |
| Reduced pH | 29.8 | 37.8 | 7.71 | 2220.2 | 1124.24 | 6/16 |
| Reduced pH | 30 | 37.8 | 7.71 | 2218.8 | 1109.74 | 6/16 |
| Reduced pH | 29.8 | 38.1 | 7.70 | 2211.5 | 1127.64 | 6/16 |
| Reduced pH | 30.3 | 37.9 | 7.70 | 2218.3 | 1154.47 | 6/16 |
| Reduced pH | 30 | 38 | 7.76 | 2249.3 | 990.72 | 6/21 |
| Reduced pH | 30 | 38.1 | 7.77 | 2251.0 | 967.05 | 6/21 |
| Reduced pH | 30.1 | 37.8 | 7.77 | 2252.2 | 980.55 | 6/21 |
| Reduced pH | 30.2 | 37.9 | 7.76 | 2250.5 | 1000.85 | 6/21 |
| Reduced pH | 30.1 | 38.2 | 7.87 | 2273.7 | 755.38 | 6/26 |
| Reduced pH | 30 | 38.2 | 7.87 | 2269.3 | 759.26 | 6/26 |
| Reduced pH | 30 | 38.2 | 7.87 | 2277.0 | 760.80 | 6/26 |
| Reduced pH | 30.2 | 38.3 | 7.88 | 2267.2 | 745.14 | 6/26 |
| Reduced pH | 30.2 | 38.2 | 7.78 | 2297.9 | 965.75 | 7/8 |
| Reduced pH | 30.2 | 38 | 7.78 | 2304.8 | 979.59 | 7/8 |
| Reduced pH | 29.8 | 38.3 | 7.80 | 2314.1 | 908.53 | 7/8 |
| Reduced pH | 29.1 | 38.2 | 7.83 | 2303.9 | 817.35 | 7/8 |
| Reduced pH | 30.4 | 38.2 | 7.74 | 2341.6 | 1117.50 | 7/11 |
| Reduced pH | 29.9 | 38.1 | 7.75 | 2334.4 | 1054.41 | 7/11 |
| Reduced pH | 29.7 | 38.1 | 7.77 | 2335.4 | 1001.19 | 7/11 |
| Reduced pH | 29.1 | 38.1 | 7.79 | 2340.8 | 917.16 | 7/11 |
| Reduced pH | 30.2 | 38.4 | 7.72 | 2266.7 | 1110.57 | 7/16 |
| Reduced pH | 30.2 | 38.3 | 7.71 | 2260.1 | 1137.99 | 7/16 |
| Reduced pH | 29.7 | 38.2 | 7.76 | 2268.1 | 992.37 | 7/16 |
| Reduced pH | 29 | 38.3 | 7.79 | 2262.1 | 888.19 | 7/16 |
| Reduced pH | 30.2 | 38 | 7.84 | 2296.7 | 827.36 | 7/27 |
| Reduced pH | 30.3 | 37.8 | 7.83 | 2289.0 | 845.33 | 7/27 |
| Reduced pH | 29.9 | 37.7 | 7.86 | 2284.4 | 767.20 | 7/27 |
| Reduced pH | 29.3 | 37.8 | 7.86 | 2278.1 | 755.15 | 7/27 |
| Reduced pH | 30.4 | 38.4 | 7.85 | 2259.0 | 792.59 | 7/30 |
| Reduced pH | 29.9 | 38.2 | 7.86 | 2259.6 | 771.67 | 7/30 |
| Reduced pH | 29.7 | 38.2 | 7.89 | 2267.8 | 711.14 | 7/30 |
| Reduced pH | 29.2 | 38.1 | 7.89 | 2269.8 | 694.22 | 7/30 |
| Reduced pH | 30.4 | 38.1 | 7.78 | 2291.5 | 970.06 | 8/4 |
| Reduced pH | 30 | 38 | 7.79 | 2288.3 | 943.24 | 8/4 |
| Reduced pH | 29.8 | 37.9 | 7.85 | 2291.7 | 797.42 | 8/4 |
| Reduced pH | 29.2 | 38 | 7.81 | 2299.7 | 871.93 | 8/4 |
| Reduced pH + Elevated Temperature | 32 | 37.5 | 7.83 | 2252.2 | 889.23 | 5/26 |
| Reduced pH + Elevated Temperature | 31.8 | 37.2 | 7.76 | 2269.1 | 1091.80 | 5/26 |
| Reduced pH + Elevated Temperature | 31.8 | 37.3 | 7.77 | 2254.5 | 1042.73 | 5/26 |
| Reduced pH + Elevated Temperature | 32 | 37.3 | 7.75 | 2255.0 | 1093.40 | 5/26 |
| Reduced pH + Elevated Temperature | 31.6 | 37.3 | 7.70 | 2304.5 | 1266.72 | 5/31 |
| Reduced pH + Elevated Temperature | 31.9 | 37.2 | 7.73 | 2304.9 | 1199.63 | 5/31 |
| Reduced pH + Elevated Temperature | 31.9 | 37.2 | 7.71 | 2309.2 | 1242.86 | 5/31 |
| Reduced pH + Elevated Temperature | 31.8 | 37.3 | 7.70 | 2313.9 | 1295.73 | 5/31 |
| Reduced pH + Elevated Temperature | 31.8 | 37.3 | 7.71 | 2296.2 | 1247.38 | 6/5 |
| Reduced pH + Elevated Temperature | 31.8 | 37 | 7.68 | 2292.0 | 1338.17 | 6/5 |
| Reduced pH + Elevated Temperature | 31.8 | 37.3 | 7.68 | 2327.1 | 1347.83 | 6/5 |
| Reduced pH + Elevated Temperature | 31.8 | 37.2 | 7.65 | 2328.0 | 1458.93 | 6/5 |
| Reduced pH + Elevated Temperature | 31.9 | 37.4 | 7.74 | 2341.5 | 1189.77 | 6/13 |
| Reduced pH + Elevated Temperature | 31.7 | 37.3 | 7.74 | 2350.4 | 1172.55 | 6/13 |
| Reduced pH + Elevated Temperature | 31.9 | 37.3 | 7.74 | 2337.1 | 1171.24 | 6/13 |
| Reduced pH + Elevated Temperature | 31.7 | 37.2 | 7.75 | 2334.1 | 1146.91 | 6/13 |
| Reduced pH + Elevated Temperature | 31.8 | 37.8 | 7.69 | 2216.2 | 1241.97 | 6/16 |
| Reduced pH + Elevated Temperature | 31.8 | 38.1 | 7.69 | 2210.3 | 1241.06 | 6/16 |
| Reduced pH + Elevated Temperature | 31.8 | 38 | 7.69 | 2213.8 | 1241.18 | 6/16 |
| Reduced pH + Elevated Temperature | 31.8 | 37.8 | 7.69 | 2213.8 | 1261.19 | 6/16 |
| Reduced pH + Elevated Temperature | 31.7 | 38.1 | 7.71 | 2271.2 | 1226.09 | 6/21 |
| Reduced pH + Elevated Temperature | 32 | 38 | 7.70 | 2263.8 | 1247.42 | 6/21 |
| Reduced pH + Elevated Temperature | 31.8 | 38.1 | 7.72 | 2250.6 | 1186.42 | 6/21 |
| Reduced pH + Elevated Temperature | 31.9 | 38.1 | 7.71 | 2253.1 | 1218.29 | 6/21 |
| Reduced pH + Elevated Temperature | 31.8 | 38.3 | 7.82 | 2274.2 | 927.67 | 6/26 |
| Reduced pH + Elevated Temperature | 31.9 | 39.3 | 7.81 | 2273.0 | 932.11 | 6/26 |
| Reduced pH + Elevated Temperature | 31.8 | 38.2 | 7.85 | 2272.6 | 856.37 | 6/26 |
| Reduced pH + Elevated Temperature | 32 | 38.4 | 7.82 | 2265.8 | 912.24 | 6/26 |
| Reduced pH + Elevated Temperature | 31.8 | 38.3 | 7.74 | 2300.1 | 1158.39 | 7/8 |
| Reduced pH + Elevated Temperature | 31.9 | 38.4 | 7.73 | 2306.0 | 1166.74 | 7/8 |
| Reduced pH + Elevated Temperature | 31.7 | 38.4 | 7.75 | 2301.5 | 1115.93 | 7/8 |
| Reduced pH + Elevated Temperature | 31.7 | 38.4 | 7.75 | 2304.1 | 1102.85 | 7/8 |
| Reduced pH + Elevated Temperature | 31.9 | 38.2 | 7.70 | 2345.9 | 1283.71 | 7/11 |
| Reduced pH + Elevated Temperature | 31.8 | 38.4 | 7.71 | 2340.3 | 1244.25 | 7/11 |
| Reduced pH + Elevated Temperature | 31.9 | 38.3 | 7.71 | 2334.4 | 1252.30 | 7/11 |
| Reduced pH + Elevated Temperature | 30.3 | 38.3 | 7.75 | 2338.5 | 1084.47 | 7/11 |
| Reduced pH + Elevated Temperature | 31.7 | 38.2 | 7.69 | 2271.7 | 1288.11 | 7/16 |
| Reduced pH + Elevated Temperature | 31.6 | 38.6 | 7.69 | 2271.9 | 1275.07 | 7/16 |
| Reduced pH + Elevated Temperature | 31.8 | 38.4 | 7.70 | 2268.1 | 1239.19 | 7/16 |
| Reduced pH + Elevated Temperature | 30.9 | 38.5 | 7.71 | 2255.2 | 1184.21 | 7/16 |
| Reduced pH + Elevated Temperature | 31.7 | 37.8 | 7.80 | 2280.6 | 963.50 | 7/27 |
| Reduced pH + Elevated Temperature | 31.9 | 38.1 | 7.80 | 2282.0 | 978.50 | 7/27 |
| Reduced pH + Elevated Temperature | 31.7 | 38 | 7.80 | 2284.8 | 964.26 | 7/27 |
| Reduced pH + Elevated Temperature | 31.9 | 38 | 7.80 | 2283.3 | 979.30 | 7/27 |
| Reduced pH + Elevated Temperature | 31.7 | 38.3 | 7.80 | 2262.3 | 955.13 | 7/30 |
| Reduced pH + Elevated Temperature | 31.8 | 38.4 | 7.80 | 2260.4 | 965.08 | 7/30 |
| Reduced pH + Elevated Temperature | 31.9 | 38.5 | 7.83 | 2265.6 | 891.16 | 7/30 |
| Reduced pH + Elevated Temperature | 31.8 | 38.4 | 7.81 | 2261.4 | 941.78 | 7/30 |
| Reduced pH + Elevated Temperature | 31.7 | 38.2 | 7.75 | 2286.7 | 1113.28 | 8/4 |
| Reduced pH + Elevated Temperature | 31.7 | 38.2 | 7.75 | 2287.7 | 1109.08 | 8/4 |
| Reduced pH + Elevated Temperature | 31.8 | 38.3 | 7.75 | 2287.6 | 1110.32 | 8/4 |
| Reduced pH + Elevated Temperature | 31 | 38.1 | 7.75 | 2288.1 | 1090.77 | 8/4 |

**Stage-III Geotaxis Data by Brood**

| Brood | Temperature | CO_2_ | Treatment | Up(%) |
| --- | --- | --- | --- | --- |
| A | Ambient | Ambient | Control | 60 |
| B | Ambient | Ambient | Control | 80 |
| C | Ambient | Ambient | Control | 60 |
| D | Ambient | Ambient | Control | 60 |
| E | Ambient | Ambient | Control | 70 |
| F | Ambient | Ambient | Control | 100 |
| G | Ambient | Ambient | Control | 40 |
| A | Elevated | Ambient | Elevated Temperature | 90 |
| B | Elevated | Ambient | Elevated Temperature | 80 |
| C | Elevated | Ambient | Elevated Temperature | 60 |
| D | Elevated | Ambient | Elevated Temperature | 70 |
| E | Elevated | Ambient | Elevated Temperature | 80 |
| F | Elevated | Ambient | Elevated Temperature | 60 |
| G | Elevated | Ambient | Elevated Temperature | 50 |
| A | Ambient | Elevated | Reduced pH | 40 |
| B | Ambient | Elevated | Reduced pH | 40 |
| C | Ambient | Elevated | Reduced pH | 20 |
| D | Ambient | Elevated | Reduced pH | 0 |
| E | Ambient | Elevated | Reduced pH | 20 |
| F | Ambient | Elevated | Reduced pH | 10 |
| G | Ambient | Elevated | Reduced pH | 20 |
| A | Elevated | Elevated | Reduced pH + Elevated Temperature | 50 |
| B | Elevated | Elevated | Reduced pH + Elevated Temperature | 10 |
| C | Elevated | Elevated | Reduced pH + Elevated Temperature | 10 |
| D | Elevated | Elevated | Reduced pH + Elevated Temperature | 0 |
| E | Elevated | Elevated | Reduced pH + Elevated Temperature | 20 |
| F | Elevated | Elevated | Reduced pH + Elevated Temperature | 40 |
| G | Elevated | Elevated | Reduced pH + Elevated Temperature | 0 |

**Stage-V Geotaxis Data by Brood**

| Brood | Treatment | Up(%) |
| --- | --- | --- |
| A | Control | 50 |
| B | Control | 40 |
| C | Control | 0 |
| D | Control | 70 |
| E | Control | 10 |
| A | Elevated Temperature | 30 |
| B | Elevated Temperature | 50 |
| C | Elevated Temperature | 0 |
| D | Elevated Temperature | 30 |
| E | Elevated Temperature | 30 |
| A | Reduced pH | 40 |
| B | Reduced pH | 20 |
| C | Reduced pH | 30 |
| D | Reduced pH | 30 |
| E | Reduced pH | 0 |
| A | Reduced pH + Elevated Temperature | 30 |
| B | Reduced pH + Elevated Temperature | 20 |
| C | Reduced pH + Elevated Temperature | 20 |
| D | Reduced pH + Elevated Temperature | 20 |
| E | Reduced pH + Elevated Temperature | 0 |

**Stage-III Geotaxis and Swimming Speed Data by Individual**

| Brood | Treatment | Direction | Speed |
| --- | --- | --- | --- |
| A | Control | up | 0.5 |
| A | Control | up | 0.31 |
| A | Control | down | -0.12 |
| A | Control | down | -0.06 |
| A | Control | down | -0.35 |
| A | Control | up | 0.26 |
| A | Control | down | -0.03 |
| A | Control | up | 0.31 |
| A | Control | up | 0.07 |
| A | Control | up | 0.15 |
| B | Control | up | 0.55 |
| B | Control | up | 0.83 |
| B | Control | up | 1.4 |
| B | Control | up | 0.56 |
| B | Control | up | 0.96 |
| B | Control | up | 0.33 |
| B | Control | up | 0.41 |
| B | Control | down | -0.13 |
| B | Control | up | 0.69 |
| B | Control | down | -0.15 |
| C | Control | down | -0.86 |
| C | Control | up | 0.43 |
| C | Control | up | 0.74 |
| C | Control | down | -0.85 |
| C | Control | up | 0.19 |
| C | Control | up | 0.57 |
| C | Control | up | 0.64 |
| C | Control | down | -0.56 |
| C | Control | down | -0.17 |
| C | Control | up | 0.67 |
| D | Control | up | 0.08 |
| D | Control | up | 0.29 |
| D | Control | up | 0.04 |
| D | Control | up | 0.05 |
| D | Control | down | -0.22 |
| D | Control | down | -0.18 |
| D | Control | up | 0.15 |
| D | Control | neutral | 0 |
| D | Control | down | -0.14 |
| D | Control | up | 0.06 |
| E | Control | up | 0.58 |
| E | Control | down | -0.13 |
| E | Control | up | 0.23 |
| E | Control | up | 0.09 |
| E | Control | down | -0.1 |
| E | Control | down | -0.17 |
| E | Control | up | 0.18 |
| E | Control | up | 0.5 |
| E | Control | up | 0.35 |
| E | Control | up | 0.06 |
| F | Control | up | 0.8 |
| F | Control | up | 0.53 |
| F | Control | up | 0.52 |
| F | Control | up | 0.63 |
| F | Control | up | 0.49 |
| F | Control | up | 0.68 |
| F | Control | up | 0.92 |
| F | Control | up | 0.98 |
| F | Control | up | 0.68 |
| F | Control | up | 0.9 |
| G | Control | down | -0.2 |
| G | Control | down | -0.22 |
| G | Control | up | 0.8 |
| G | Control | down | -0.15 |
| G | Control | up | 0.15 |
| G | Control | down | -0.14 |
| G | Control | up | 0.18 |
| G | Control | neutral | 0 |
| G | Control | down | -0.05 |
| G | Control | up | 0.23 |
| A | Elevated Temperature | up | 0.03 |
| A | Elevated Temperature | up | 0.3 |
| A | Elevated Temperature | up | 0.62 |
| A | Elevated Temperature | up | 0.15 |
| A | Elevated Temperature | up | 0.31 |
| A | Elevated Temperature | up | 0.12 |
| A | Elevated Temperature | neutral | 0 |
| A | Elevated Temperature | up | 0.21 |
| A | Elevated Temperature | up | 0.36 |
| A | Elevated Temperature | up | 0.26 |
| B | Elevated Temperature | down | -0.52 |
| B | Elevated Temperature | up | 0.51 |
| B | Elevated Temperature | up | 0.04 |
| B | Elevated Temperature | down | -0.34 |
| B | Elevated Temperature | up | 0.35 |
| B | Elevated Temperature | up | 0.1 |
| B | Elevated Temperature | up | 0.08 |
| B | Elevated Temperature | up | 0.27 |
| B | Elevated Temperature | up | 0.4 |
| B | Elevated Temperature | up | 0.35 |
| C | Elevated Temperature | down | -0.71 |
| C | Elevated Temperature | down | -0.1 |
| C | Elevated Temperature | up | 0.68 |
| C | Elevated Temperature | down | -0.56 |
| C | Elevated Temperature | up | 0.29 |
| C | Elevated Temperature | down | -0.25 |
| C | Elevated Temperature | up | 0.16 |
| C | Elevated Temperature | up | 0.36 |
| C | Elevated Temperature | up | 0.25 |
| C | Elevated Temperature | up | 0.42 |
| D | Elevated Temperature | down | -0.08 |
| D | Elevated Temperature | up | 0.38 |
| D | Elevated Temperature | up | 0.1 |
| D | Elevated Temperature | down | -0.1 |
| D | Elevated Temperature | up | 0.14 |
| D | Elevated Temperature | up | 0.25 |
| D | Elevated Temperature | up | 0.08 |
| D | Elevated Temperature | down | -0.07 |
| D | Elevated Temperature | up | 0.3 |
| D | Elevated Temperature | up | 0.12 |
| E | Elevated Temperature | down | -0.22 |
| E | Elevated Temperature | up | 0.86 |
| E | Elevated Temperature | down | -0.16 |
| E | Elevated Temperature | up | 0.38 |
| E | Elevated Temperature | up | 0.07 |
| E | Elevated Temperature | up | 0.09 |
| E | Elevated Temperature | up | 0.16 |
| E | Elevated Temperature | up | 0.18 |
| E | Elevated Temperature | up | 0.06 |
| E | Elevated Temperature | up | 0.42 |
| F | Elevated Temperature | up | 0.35 |
| F | Elevated Temperature | down | -0.3 |
| F | Elevated Temperature | down | -0.5 |
| F | Elevated Temperature | up | 0.83 |
| F | Elevated Temperature | up | 0.1 |
| F | Elevated Temperature | up | 0.16 |
| F | Elevated Temperature | down | -0.11 |
| F | Elevated Temperature | up | 0.6 |
| F | Elevated Temperature | up | 0.3 |
| F | Elevated Temperature | down | -0.7 |
| G | Elevated Temperature | up | 0.22 |
| G | Elevated Temperature | down | -0.56 |
| G | Elevated Temperature | up | 0.3 |
| G | Elevated Temperature | neutral | 0 |
| G | Elevated Temperature | up | 0.28 |
| G | Elevated Temperature | up | 0.32 |
| G | Elevated Temperature | down | -0.33 |
| G | Elevated Temperature | down | -0.43 |
| G | Elevated Temperature | down | -0.31 |
| G | Elevated Temperature | up | 0.24 |
| A | Reduced pH | down | -0.44 |
| A | Reduced pH | up | 0.28 |
| A | Reduced pH | up | 0.3 |
| A | Reduced pH | up | 0.2 |
| A | Reduced pH | up | 0.3 |
| A | Reduced pH | down | -0.27 |
| A | Reduced pH | down | -0.28 |
| A | Reduced pH | down | -0.37 |
| A | Reduced pH | down | -0.03 |
| A | Reduced pH | down | -0.27 |
| B | Reduced pH | down | -0.2 |
| B | Reduced pH | down | -0.34 |
| B | Reduced pH | down | -0.5 |
| B | Reduced pH | down | -0.31 |
| B | Reduced pH | up | 0.7 |
| B | Reduced pH | down | -0.22 |
| B | Reduced pH | up | 0.6 |
| B | Reduced pH | up | 0.8 |
| B | Reduced pH | neutral | 0 |
| B | Reduced pH | up | 0.78 |
| C | Reduced pH | down | -1.13 |
| C | Reduced pH | down | -1 |
| C | Reduced pH | up | 0.4 |
| C | Reduced pH | down | -0.81 |
| C | Reduced pH | up | 0.67 |
| C | Reduced pH | down | -0.8 |
| C | Reduced pH | down | -0.77 |
| C | Reduced pH | down | -0.7 |
| C | Reduced pH | down | -1.12 |
| C | Reduced pH | down | -0.87 |
| D | Reduced pH | down | -1 |
| D | Reduced pH | down | -0.64 |
| D | Reduced pH | down | -0.5 |
| D | Reduced pH | down | -0.36 |
| D | Reduced pH | down | -0.31 |
| D | Reduced pH | down | -0.26 |
| D | Reduced pH | down | -0.28 |
| D | Reduced pH | neutral | 0 |
| D | Reduced pH | down | -0.53 |
| D | Reduced pH | down | -0.49 |
| E | Reduced pH | down | -0.68 |
| E | Reduced pH | up | 0.25 |
| E | Reduced pH | down | -0.7 |
| E | Reduced pH | down | -0.68 |
| E | Reduced pH | down | -0.61 |
| E | Reduced pH | down | -0.73 |
| E | Reduced pH | down | -0.73 |
| E | Reduced pH | down | -0.75 |
| E | Reduced pH | up | 0.31 |
| E | Reduced pH | down | -0.64 |
| F | Reduced pH | down | -0.27 |
| F | Reduced pH | down | -0.53 |
| F | Reduced pH | down | -0.49 |
| F | Reduced pH | down | -0.44 |
| F | Reduced pH | down | -0.34 |
| F | Reduced pH | down | -0.24 |
| F | Reduced pH | down | -0.31 |
| F | Reduced pH | down | -0.12 |
| F | Reduced pH | up | 0.71 |
| F | Reduced pH | down | -0.34 |
| G | Reduced pH | neutral | 0 |
| G | Reduced pH | down | -0.56 |
| G | Reduced pH | down | -0.62 |
| G | Reduced pH | down | -0.56 |
| G | Reduced pH | down | -0.79 |
| G | Reduced pH | down | -0.73 |
| G | Reduced pH | up | 0.35 |
| G | Reduced pH | down | -0.46 |
| G | Reduced pH | down | -0.64 |
| G | Reduced pH | up | 0.25 |
| A | Elevated Temperature + Reduced pH | up | 0.1 |
| A | Elevated Temperature + Reduced pH | up | 0.21 |
| A | Elevated Temperature + Reduced pH | up | 0.3 |
| A | Elevated Temperature + Reduced pH | down | -0.24 |
| A | Elevated Temperature + Reduced pH | down | -0.21 |
| A | Elevated Temperature + Reduced pH | down | -0.27 |
| A | Elevated Temperature + Reduced pH | up | 0.06 |
| A | Elevated Temperature + Reduced pH | up | 0.23 |
| A | Elevated Temperature + Reduced pH | down | -0.35 |
| A | Elevated Temperature + Reduced pH | down | -0.13 |
| B | Elevated Temperature + Reduced pH | down | -0.29 |
| B | Elevated Temperature + Reduced pH | down | -0.43 |
| B | Elevated Temperature + Reduced pH | down | -0.5 |
| B | Elevated Temperature + Reduced pH | down | -0.49 |
| B | Elevated Temperature + Reduced pH | down | -0.47 |
| B | Elevated Temperature + Reduced pH | up | 0.1 |
| B | Elevated Temperature + Reduced pH | down | -0.53 |
| B | Elevated Temperature + Reduced pH | down | -0.51 |
| B | Elevated Temperature + Reduced pH | down | -0.6 |
| B | Elevated Temperature + Reduced pH | down | -0.61 |
| C | Elevated Temperature + Reduced pH | down | -0.91 |
| C | Elevated Temperature + Reduced pH | down | -0.84 |
| C | Elevated Temperature + Reduced pH | down | -0.57 |
| C | Elevated Temperature + Reduced pH | down | -0.6 |
| C | Elevated Temperature + Reduced pH | down | -0.3 |
| C | Elevated Temperature + Reduced pH | up | 0.1 |
| C | Elevated Temperature + Reduced pH | down | -0.33 |
| C | Elevated Temperature + Reduced pH | down | -0.41 |
| C | Elevated Temperature + Reduced pH | down | -0.66 |
| C | Elevated Temperature + Reduced pH | down | -0.52 |
| D | Elevated Temperature + Reduced pH | down | -0.88 |
| D | Elevated Temperature + Reduced pH | down | -0.58 |
| D | Elevated Temperature + Reduced pH | down | -0.54 |
| D | Elevated Temperature + Reduced pH | down | -0.39 |
| D | Elevated Temperature + Reduced pH | down | -0.5 |
| D | Elevated Temperature + Reduced pH | down | -0.25 |
| D | Elevated Temperature + Reduced pH | down | -0.5 |
| D | Elevated Temperature + Reduced pH | down | -0.59 |
| D | Elevated Temperature + Reduced pH | neutral | 0 |
| D | Elevated Temperature + Reduced pH | down | -0.64 |
| E | Elevated Temperature + Reduced pH | up | 0.05 |
| E | Elevated Temperature + Reduced pH | down | -0.64 |
| E | Elevated Temperature + Reduced pH | down | -0.68 |
| E | Elevated Temperature + Reduced pH | down | -0.59 |
| E | Elevated Temperature + Reduced pH | down | -0.29 |
| E | Elevated Temperature + Reduced pH | down | -0.35 |
| E | Elevated Temperature + Reduced pH | down | -0.19 |
| E | Elevated Temperature + Reduced pH | up | 0.44 |
| E | Elevated Temperature + Reduced pH | down | -0.35 |
| E | Elevated Temperature + Reduced pH | down | -0.67 |
| F | Elevated Temperature + Reduced pH | down | -0.21 |
| F | Elevated Temperature + Reduced pH | down | -0.5 |
| F | Elevated Temperature + Reduced pH | up | 0.03 |
| F | Elevated Temperature + Reduced pH | down | -0.63 |
| F | Elevated Temperature + Reduced pH | up | 0.19 |
| F | Elevated Temperature + Reduced pH | down | -0.48 |
| F | Elevated Temperature + Reduced pH | up | 0.43 |
| F | Elevated Temperature + Reduced pH | up | 0.03 |
| F | Elevated Temperature + Reduced pH | down | -0.33 |
| F | Elevated Temperature + Reduced pH | down | -0.27 |
| G | Elevated Temperature + Reduced pH | down | -0.43 |
| G | Elevated Temperature + Reduced pH | down | -0.52 |
| G | Elevated Temperature + Reduced pH | down | -0.27 |
| G | Elevated Temperature + Reduced pH | down | -0.39 |
| G | Elevated Temperature + Reduced pH | down | -0.18 |
| G | Elevated Temperature + Reduced pH | neutral | 0 |
| G | Elevated Temperature + Reduced pH | down | -0.4 |
| G | Elevated Temperature + Reduced pH | down | -0.51 |
| G | Elevated Temperature + Reduced pH | down | -0.5 |
| G | Elevated Temperature + Reduced pH | down | -0.5 |

**Stage-V Geotaxis and Swimming Speed Data by Individual**

| Brood | Treatment | Direction | Speed |
| --- | --- | --- | --- |
| A | Control | up | 0.53 |
| A | Control | up | 0.07 |
| A | Control | down | -0.13 |
| A | Control | down | -0.14 |
| A | Control | up | 0.14 |
| A | Control | up | 0.46 |
| A | Control | up | 0.28 |
| A | Control | down | -0.17 |
| A | Control | down | -0.08 |
| A | Control | down | -0.11 |
| B | Control | down | -0.97 |
| B | Control | up | 0.82 |
| B | Control | up | 0.26 |
| B | Control | down | -0.51 |
| B | Control | down | -0.37 |
| B | Control | down | -0.54 |
| B | Control | up | 0.4 |
| B | Control | down | -0.09 |
| B | Control | down | -0.58 |
| B | Control | up | 0.5 |
| C | Control | down | -0.38 |
| C | Control | down | -0.53 |
| C | Control | down | -0.32 |
| C | Control | down | -0.78 |
| C | Control | down | -0.89 |
| C | Control | down | -1.01 |
| C | Control | down | -1 |
| C | Control | down | -0.83 |
| C | Control | down | -0.78 |
| C | Control | down | -0.94 |
| D | Control | up | 0.19 |
| D | Control | up | 0.27 |
| D | Control | up | 0.5 |
| D | Control | down | -0.45 |
| D | Control | up | 0.03 |
| D | Control | up | 0.38 |
| D | Control | down | -1.04 |
| D | Control | down | -1.07 |
| D | Control | up | 0.2 |
| D | Control | up | 0.3 |
| E | Control | down | -0.26 |
| E | Control | down | -0.27 |
| E | Control | down | -0.3 |
| E | Control | down | -0.73 |
| E | Control | down | -0.29 |
| E | Control | down | -0.23 |
| E | Control | down | -0.28 |
| E | Control | down | -0.37 |
| E | Control | up | 0.2 |
| E | Control | down | -0.35 |
| A | Elevated Temperature | down | -0.36 |
| A | Elevated Temperature | up | 0.9 |
| A | Elevated Temperature | down | -0.32 |
| A | Elevated Temperature | up | 0.23 |
| A | Elevated Temperature | up | 0.17 |
| A | Elevated Temperature | down | -0.13 |
| A | Elevated Temperature | down | -0.17 |
| A | Elevated Temperature | down | -0.41 |
| A | Elevated Temperature | down | -0.39 |
| A | Elevated Temperature | down | -0.55 |
| B | Elevated Temperature | down | -0.42 |
| B | Elevated Temperature | down | -0.51 |
| B | Elevated Temperature | down | -0.43 |
| B | Elevated Temperature | down | -0.68 |
| B | Elevated Temperature | up | 0.5 |
| B | Elevated Temperature | up | 0.42 |
| B | Elevated Temperature | up | 0.06 |
| B | Elevated Temperature | up | 0.06 |
| B | Elevated Temperature | up | 0.48 |
| B | Elevated Temperature | down | -0.12 |
| C | Elevated Temperature | down | -0.82 |
| C | Elevated Temperature | down | -0.72 |
| C | Elevated Temperature | down | -0.77 |
| C | Elevated Temperature | down | -0.92 |
| C | Elevated Temperature | down | -0.77 |
| C | Elevated Temperature | down | -0.63 |
| C | Elevated Temperature | down | -0.82 |
| C | Elevated Temperature | down | -0.79 |
| C | Elevated Temperature | down | -0.78 |
| C | Elevated Temperature | down | -0.96 |
| D | Elevated Temperature | up | 0.02 |
| D | Elevated Temperature | down | -0.35 |
| D | Elevated Temperature | down | -0.11 |
| D | Elevated Temperature | down | -0.22 |
| D | Elevated Temperature | down | -0.45 |
| D | Elevated Temperature | up | 0.1 |
| D | Elevated Temperature | down | -0.11 |
| D | Elevated Temperature | down | -0.33 |
| D | Elevated Temperature | down | -0.09 |
| D | Elevated Temperature | up | 0.11 |
| E | Elevated Temperature | down | -1.18 |
| E | Elevated Temperature | down | -0.73 |
| E | Elevated Temperature | down | -0.25 |
| E | Elevated Temperature | down | -1.17 |
| E | Elevated Temperature | up | 0.87 |
| E | Elevated Temperature | down | -0.74 |
| E | Elevated Temperature | up | 0.06 |
| E | Elevated Temperature | up | 0.09 |
| E | Elevated Temperature | down | -0.7 |
| E | Elevated Temperature | down | -0.64 |
| A | Reduced pH | up | 0.31 |
| A | Reduced pH | down | -0.68 |
| A | Reduced pH | down | -0.38 |
| A | Reduced pH | down | -0.8 |
| A | Reduced pH | down | -0.5 |
| A | Reduced pH | up | 0.15 |
| A | Reduced pH | up | 1.04 |
| A | Reduced pH | up | 0.27 |
| A | Reduced pH | down | -0.42 |
| A | Reduced pH | down | -0.5 |
| B | Reduced pH | up | 0.23 |
| B | Reduced pH | up | 0.39 |
| B | Reduced pH | down | -0.44 |
| B | Reduced pH | down | -0.73 |
| B | Reduced pH | down | -0.41 |
| B | Reduced pH | down | -0.21 |
| B | Reduced pH | down | -0.33 |
| B | Reduced pH | down | -0.66 |
| B | Reduced pH | down | -0.05 |
| B | Reduced pH | down | -0.66 |
| C | Reduced pH | up | 0.8 |
| C | Reduced pH | down | -0.25 |
| C | Reduced pH | down | -0.39 |
| C | Reduced pH | down | -0.19 |
| C | Reduced pH | down | -0.2 |
| C | Reduced pH | down | -0.05 |
| C | Reduced pH | up | 0.18 |
| C | Reduced pH | up | 0.15 |
| C | Reduced pH | down | -0.17 |
| C | Reduced pH | down | -0.45 |
| D | Reduced pH | up | 1.57 |
| D | Reduced pH | down | -0.11 |
| D | Reduced pH | down | -0.03 |
| D | Reduced pH | down | -0.92 |
| D | Reduced pH | down | -0.9 |
| D | Reduced pH | up | 0.05 |
| D | Reduced pH | down | -0.53 |
| D | Reduced pH | down | -0.8 |
| D | Reduced pH | up | 0.42 |
| D | Reduced pH | down | -0.36 |
| E | Reduced pH | down | -1.02 |
| E | Reduced pH | down | -0.87 |
| E | Reduced pH | down | -0.9 |
| E | Reduced pH | down | -1.15 |
| E | Reduced pH | down | -0.9 |
| E | Reduced pH | down | -0.67 |
| E | Reduced pH | down | -1.04 |
| E | Reduced pH | down | -0.9 |
| E | Reduced pH | down | -1.38 |
| E | Reduced pH | down | -0.78 |
| A | Elevated Temperature + Reduced pH | up | 0.13 |
| A | Elevated Temperature + Reduced pH | up | 0.19 |
| A | Elevated Temperature + Reduced pH | down | -0.47 |
| A | Elevated Temperature + Reduced pH | down | -0.7 |
| A | Elevated Temperature + Reduced pH | down | -0.03 |
| A | Elevated Temperature + Reduced pH | up | 0.93 |
| A | Elevated Temperature + Reduced pH | down | -0.47 |
| A | Elevated Temperature + Reduced pH | down | -0.78 |
| A | Elevated Temperature + Reduced pH | down | -0.1 |
| A | Elevated Temperature + Reduced pH | down | -0.6 |
| B | Elevated Temperature + Reduced pH | down | -0.63 |
| B | Elevated Temperature + Reduced pH | down | -0.21 |
| B | Elevated Temperature + Reduced pH | up | 0.09 |
| B | Elevated Temperature + Reduced pH | down | -0.38 |
| B | Elevated Temperature + Reduced pH | down | -0.17 |
| B | Elevated Temperature + Reduced pH | down | -0.29 |
| B | Elevated Temperature + Reduced pH | down | -0.13 |
| B | Elevated Temperature + Reduced pH | up | 0.88 |
| B | Elevated Temperature + Reduced pH | down | -0.4 |
| B | Elevated Temperature + Reduced pH | down | -1.32 |
| C | Elevated Temperature + Reduced pH | down | -0.27 |
| C | Elevated Temperature + Reduced pH | up | 0.65 |
| C | Elevated Temperature + Reduced pH | down | -0.22 |
| C | Elevated Temperature + Reduced pH | down | -0.63 |
| C | Elevated Temperature + Reduced pH | down | -0.44 |
| C | Elevated Temperature + Reduced pH | down | -0.15 |
| C | Elevated Temperature + Reduced pH | down | -0.33 |
| C | Elevated Temperature + Reduced pH | down | -0.41 |
| C | Elevated Temperature + Reduced pH | down | -0.16 |
| C | Elevated Temperature + Reduced pH | up | 0.26 |
| D | Elevated Temperature + Reduced pH | down | -0.35 |
| D | Elevated Temperature + Reduced pH | up | 0.48 |
| D | Elevated Temperature + Reduced pH | down | -0.5 |
| D | Elevated Temperature + Reduced pH | up | 0.22 |
| D | Elevated Temperature + Reduced pH | down | -0.5 |
| D | Elevated Temperature + Reduced pH | down | -0.34 |
| D | Elevated Temperature + Reduced pH | down | -0.48 |
| D | Elevated Temperature + Reduced pH | down | -0.47 |
| D | Elevated Temperature + Reduced pH | down | -0.49 |
| D | Elevated Temperature + Reduced pH | down | -0.89 |
| E | Elevated Temperature + Reduced pH | down | -0.42 |
| E | Elevated Temperature + Reduced pH | down | -0.9 |
| E | Elevated Temperature + Reduced pH | down | -0.75 |
| E | Elevated Temperature + Reduced pH | down | -0.84 |
| E | Elevated Temperature + Reduced pH | down | -0.48 |
| E | Elevated Temperature + Reduced pH | down | -0.8 |
| E | Elevated Temperature + Reduced pH | down | -0.58 |
| E | Elevated Temperature + Reduced pH | down | -0.31 |
| E | Elevated Temperature + Reduced pH | down | -0.79 |
| E | Elevated Temperature + Reduced pH | down | -0.62 |
